# Evaluation of the mechanism of Gong Ying San activity on dairy cows mastitis by network pharmacology and metabolomics analysis

**DOI:** 10.1371/journal.pone.0299234

**Published:** 2024-04-17

**Authors:** Shuang Gao, Liyun Tang, Jiayi Ma, Kaiming Wang, Hua Yao, Jinjin Tong, Hua Zhang

**Affiliations:** 1 Animal Science and Technology College, Beijing University of Agriculture, Beijing, P.R. China; 2 Department of Biomedical Engineering, The Hong Kong Polytechnic University, Hong Kong, P.R. China; Alexandria University, EGYPT

## Abstract

**Objectives:**

The goal of this investigation was to identify the main compounds and the pharmacological mechanism of the traditional Chinese medicine formulation, Gong Ying San (GYS), by infrared spectral absorption characteristics, metabolomics, network pharmacology, and molecular-docking analysis for mastitis. The antibacterial and antioxidant activities were determined in vitro.

**Methods:**

The chemical constituents of GYS were detected by ultra-high-performance liquid chromatography Q-extractive mass spectrometry (UHPLC-QE-MS). Related compounds were screened from the Traditional Chinese Medicine Systems Pharmacology Database and Analysis Platform (TCMSP, http://tcmspw.com/tcmsp.php) and the Encyclopedia of Traditional Chinese Medicine (ETCM, http://www.tcmip.cn/ETCM/index.php/Home/) databases; genes associated with mastitis were identified in DisGENT. A protein-protein interaction (PPI) network was generated using STRING. Gene ontology (GO) and Kyoto Encyclopedia of Genes and Genomes (KEGG) pathway enrichment screening was conducted using the R module. Molecular-docking analyses were performed with the AutoDockTools V1.5.6.

**Results:**

Fifty-four possible compounds in GYS with forty likely targets were found. The compound-target-network analysis showed that five of the ingredients, quercetin, luteolin, kaempferol, beta-sitosterol, and stigmasterol, had degree values >41.6, and the genes *TNF*, *IL-6*, *IL-1β*, *ICAM1*, *CXCL8*, *CRP*, *IFNG*, *TP53*, *IL-2*, and *TGFB1* were core targets in the network. Enrichment analysis revealed that pathways associated with cancer, lipids, atherosclerosis, and PI3K-Akt signaling pathways may be critical in the pharmacology network. Molecular-docking data supported the hypothesis that quercetin and luteolin interacted well with TNF-α and IL-6.

**Conclusions:**

An integrative investigation based on a bioinformatics-network topology provided new insights into the synergistic, multicomponent mechanisms of GYS’s anti-inflammatory, antibacterial, and antioxidant activities. It revealed novel possibilities for developing new combination medications for reducing mastitis and its complications.

## Introduction

Mastitis is a complex disease with imperfectly known etiology. As dairy cows’ most widespread infectious disease, it harms animal health, reduces milk quality, and represents a global economic problem for milk producers [1]. The susceptibility to and development of mastitis is associated with the degree to which mammary glands suffer exposure to pathogenic bacteria. How severe the infection will be depends on a number of host-specific elements, such as nutrient intake, genetic susceptibility, oxidant levels, and the changes in mammary tissues associated with the switch from involution to lactation [2]. *Escherichia coli*, *Staphylococcus aureus*, *Streptococcus agalactiae*, *and Klebsiella pneumoniae* are the main pathogenic bacteria in bovine mastitis [3]. Current mastitis treatments include antibiotics [4], antimicrobial peptides [5], bacteriophage therapy [6], probiotics [7], and nanoparticle-based treatments [8]. The broad effectiveness of antibiotics makes them the most popular therapy. However, overreliance on antibiotics contributes to the development of multidrug-resistant bacteria [9]. Because of this disadvantage, exploring traditional Chinese medicine (TCM) for practical, green formulas that can be used in place of antibiotics is worthwhile. The discovery of safe, efficient antibacterial or anti-mastitis drugs for use by dairy farmers in milk production is urgently needed. TCM has been receiving more attention and scrutiny worldwide for providing new options.

Gong Ying San (GYS) is a classic TCM herbal formulation, composed of Zhe Bei Mu (*Fritillariae thunbergii* bulbus), Jin Yin Hua (*Lonicerae japonicae flos*), Lian Qiao (*Forsythiae fructus*), Tong Cao (*Medulla tetrapanacis*), Si Gua Lao (*loofah*) and Pu Gong Ying (*dandelion*) that has been widely used for mastitis in dairy cows in China [10]. GYS has been recognized as exerting clearing heat and detoxification, eliminating redness and swelling in acute and chronic inflammatory conditions. The alkaloid and flavonoid compounds contained in GYS can play a resistance role in the pathogenic bacteria causing bovine mastitis, promoting the formation of lymphocytes in the body, enhancing immune capability, preventing the occurrence and development of inflammation, promoting the growth of mammary epithelial cells, enhancing the metabolic capacity of the mammary tissue and encouraging milk secretion [11]. A previous study indicated that dandelion, one of GYS’s main ingredients, could alleviate LPS-induced oxidant stress by scavenging reactive oxygen species (ROS); the compound promoted antioxidant enzyme activity by up-regulating gene expression through the Nrf2 signaling pathwa/y [12]. Anti-inflammatory effects are an essential pharmacological activity of *Fritillariae thunbergii* bulbus, inhibiting the immunological response of T lymphocytes and reducing the NF-κB pathway [13]. *Lonicerae Japonicae Flos* and *Forsythiae Fructus* can inhibit the expression of α-SMA, COX2, FPR2, PTGS1, NCOA2, IL-1β,TNF-α, CXCL14, and TGF-β1and reduce inflammatory responses and cellular oxidant levels [14]. Moreover, it was shown that an aqueous extract of *Medulla tetrapanacis* possessed anti-inflammatory and bactericidal effects and prevented polarization of macrophages, which reduced the level of inflammatory mediators and phagocytosis inactivating the MAPK pathway. It also inhibited the growth of *S*. *aureus* [15]. Treatments with a course of antibiotics are expensive, and overuse of antibiotics may increase the number of humans and animals infected by antibiotic-resistant bacteria [16]. Interestingly, feeding GYS powder to dairy cows with subclinical mastitis significantly improved milk quality, reduced the somatic cell count (SCC), and increased milk yield in mid-lactation dairy cows compared to conventional antibiotic treatment [17]. Although several researchers have studied the anti-inflammatory effect of GYS and its components in dairy cows [18, 19], the underlying comprehensive pharmacological mechanism of GYS’s action has not been elucidated because of its complex composition and the problem of determining the several targets of TCM formulations.

Network pharmacology, which integrates multi-omics, is a useful technique for studying the activities and functional pathways of TCM formulations. It provides a novel strategy for determining the network of TCM-compound-protein/gene-disease interactions and the primary active molecules with therapeutic properties [20]. Network pharmacology methods differ from those of the conventional one-target-one-drug approach. This analytical tool targets the diversity of interactions in living systems between the active compounds and the pathophysiological conditions associated with the disease [1]. Molecular docking is a widely accepted technique utilizing a computer-generated molecular structure model, with which we can deduce the interactions between active components and physiological systems [21]. Studies have shown that molecular-docking assays can measure the binding strength between the drugs and critical targets, improving the molecular network’s accuracy [22].

In this study, we used metabolomics technology to determine the chemical composition of GYS. Network pharmacology software and databases were employed to pinpoint the likeliest targets and biological pathways of the active components of GYS in dairy cow mastitis. Molecular docking were used to determine the interaction of potential gene targets and to identify the principle compounds and the mechanism of GYS activity in mastitis. We also determined the antibacterial and antioxidant capacity of GYS, further supporting the results obtained by network pharmacology. These results will help in screening potential mastitis drugs, improving the formulation of GYS, and providing new insights into ways to improve dairy cow health by preventing mastitis.

## Materials and methods

### Sample preparation

GYS was obtained from Beijing Tongrentang Co., Ltd, China, and consisted of dandelion 60.00 g, *Lonicerae japonicae flos* 60.00 g, *Forsythiae fructus* 60.00 g, loofah 30.00 g, *Tetrapanacis medulla* 25.00 g, cottonrose hibiscus leaf 25.00g, and *Fritillariae thunbrgii bulbus* 30.00 g. A portion of GYS was powdered with a milling machine (HBM-109, Hanbo Electromechanical Co., Ltd, China), and kept sealed for later use. The powder was placed in extraction medium (ultrapure water) for 30 min, then extracted in an ultrasonic extraction instrument (KQ-700DE, Kun Shan Ultrasonic Instruments Co., Ltd, China) at 35°C for 60 min. The mixture was cooled to 25°C, centrifuged at 999 g for 15 min. The supernatant is stored at 4°C and prepared into freeze-dried powder, which can be stored at -20°C and sealed for a long time. To prepare a GYS stock solution for later analysis, 5.76 g of lyophilized GYS powder was dissolved in 10 mL of ultrapure water.

### Metabolomics profiling using ultra-high-performance liquid chromatography with Q-extractive mass spectrometry (UHPLC-QE-MS)

An aqueous extract of GYS was centrifuged at 15,984 g for fifteen minutes at 4°C, and 300 μL of supernatant was mixed with 1 mL of extract buffer containing 10 μg/mL of internal standard. Tubes were vortexed for 30 s and sonicated for five minutes at 4°C. After one hour at -40°C, the samples were re-centrifuged at 15,984g for fifteen minutes at 4°C. The supernatants were passed through a 0.22 μm filter and analyzed by UHPLC-MS/MS with QC samples (100 μL) included periodically to monitor performance. The UHPLC-MS/MS separation step was carried out with a high-performance liquid chromatograph (Vanquish, Thermo-Fisher Scientific Inc., Germany). For chromatography, a UHPLC BEH-C18 column (1.7 μm × 2.1 × 100 mm) from (Watsch/Waters Tech Co., Ltd, Shanghai, China) was used for separations. The injection volume was 5 μL, and the flow rate was 0.4 mL/min. The optimal mobile phase consisted of 0.1% formic acid in water (A) and 0.1% formic acid in acetonitrile (B). The multi-step linear elution gradient program was as follows: 0–3.5 min, 95–85% A; 3.5–6 min, 85–70% A; 6–6.5 min, 70–70% A; 6.5–12 min, 70–30% A; 12–12.5 min, 30–30% A.

An Orbitrap Exploris 120 mass spectrometer (Orbitrap Exploris 120, Thermo-Fisher Scientific Inc., Germany) equipped with Xcalibur software was used to acquire MS and MS/MS data based in the IDA-acquisition mode. The sheath-gas flow rate was 30 Arb, the auxiliary gas flow rate was 10 Arb, the capillary T was 350°C, the full millisecond resolution was 60,000, the MS/MS resolution was 15,000, the collision energy was 16/38/42 in NCE mode, and the spray V was 5.5 kV (positive) or -4 kV (negative). The caloric value standard for screening effective metabolites was VIP>1, *p*<0.05.

### Screening GYS for bioactive compounds

The TCM pharmacological database (TCMSP) and platform was employed to determine the most likely active components [23, 24]. Accordingly, two indicators, oral bioavailability (OB) and drug likeness (DL), were selected. OB is the rate of absorption and appearance of a drug in the blood after administration. DL refers to the degree to which compounds are similar to drugs already on the market [25]. An OB of ≥ 30% [26] and DL ≥ 0.18 [27] were chosen as threshold values for selecting the core active components for screening GYS.

## Prediction of candidate targets of GYS in inflammatory disease

The names of the target proteins were translated into their corresponding gene symbols with the UniProt dbase (https://www.uniprot.org) [28]; ‘mastitis’ was set as the search term to collect related targets from the DisGENT (https://www.disgenet.org/) database [29] with *Bos taurus* as the designated species. The final group of hits was acquired after deleting repetitions; the selected GYS targets and mastitis-related hits were depicted as a Venn diagram (https://bioinfogp.cnb.csic.es/tools/venny/) to illustrate the inter-relationship of specific targets with the active compounds. Lastly, these common targets were organized for subsequent network analyses.

### Functional enrichment analysis

The likely GYS targets for mastitis treatment were uploaded to the database for annotation, visualization, and integrated discovery (DAVID, https://david.ncifcrf.gov/) [30]. Then, GO and KEGG analyses were performed to identify the essential signaling pathways in mastitis treatment. The output was ranked according to the top scores of GO and KEGG evaluations. A *p*< 0.05 was used to define a significant difference between means.

### Construction of the protein-protein interaction (PPI) network

The overlapping genes interacting with GYS ingredients and inflammatory pathways were accepted as hub genes. The GYS-target-mastitis network was constructed by Cytoscape 3.9.1 software, an open-source platform for depicting network complexity and integrating multiple groups of information for analysis. The hub gene names were input to the STRING database (https://string-db.org/) after setting the combination score to >0.9 to construct the PPI network. The data were obtained as a TSV file and imported into Cytoscape V3.9.1 for further analysis. CytoHub, a Cytoscape V3.9.1 module, was employed to determine the degree of centrality. The most prominent protein targets involved in the most biological pathways were considered the most promising therapeutic targets.

### Construction of the GYS active component-target network

The screened active components and target information were uploaded to Cytoscape V3.9.1 to generate the compounds-targets-diseases (CTD) network to better display the interactions between functional components and targets. ‘Nodes’ represent drugs, compounds, and targets. ‘Edges’ show the association between one node and another. The correlation degree of nodes was determined based on the degree value.

### Molecular docking

Molecular docking capability was assessed using AutoDockTools V1.5.6 to confirm the interaction of compounds with targets [31]. The top two core targets were evaluated to verify the method’s reliability, and receptor proteins were selected. The core protein crystal structures were obtained from the protein data bank (PDB, http://www.rcsb.org/), and the PyMol program was used to optimize the protein structures by deleting H_2_O. Hydrogens were inserted into the proteins, and the resulting charge was calculated with AutoDockTools V1.5.6 and exported as a PDBQT file. The molecular docking results were presented as docking scores; the higher the score, the greater the likelihood that a protein was a target of a component from the GYS formulation.

### Antibacterial activity

*E*. *coli* CVCC 1450, *S*. *agalactiae* CVCC 3940, and *S*. *aureus* CVCC 2257 were obtained from the National Center for Veterinary Culture Collection (CVCC). Microdilution assays were used as previously described to calculate minimum inhibitory concentration (MIC) [32]. Briefly, serial two-fold dilutions of GYS extract ranging from 288 mg/mL to 0.56 mg/mL were incubated with the test bacterial cultures seeded in a 96-well plate at 3–6×10^6^ CFU/mL. The bacterial cultures were incubated with the different concentrations of GYS extract at a ratio of 1:1 (v/v) for 24 h at 37°C. The positive control was 0.2 mL of bacterial suspension with no GYS and the negative control was 0.2 mL of medium alone. The absorbance at 492 nm was measured using a microplate spectrophotometer (Tecan GENios F129004, Austria). The minimum inhibitory concentration (MIC) values were defined as the lowest concentration of GYS extract that resulted in no increase in A492. In addition to determining the MIC, the minimum bactericidal concentration (MBC), the lowest concentration of GYS that resulted in the death of 99.9% of the bacteria, was measured. Aliquots of 50 μL from all tubes with no visible bacterial growth were seeded on agar plates and incubated for 24 h at 37°C. The lowest concentration of an antimicrobial agent killing 99.9% of a bacterial population is termed the minimum bactericidal concentration (MBC). This was done by observing pre- and post-incubated agar plates for the presence or absence of bacteria. Each experiment was repeated three times.

### Disk diffusion bacteriostatic assay

For the Kirby-Bauer disc diffusion method, 0.1 mL (3–6 ×10^6^ CFU/ml) of bacterial suspension was uniformly spread on nutrient agar. Afterwards, filter paper disks (6 mm diam.) containing the antibiotics, penicillin, cephalexin, vancomycin, and gentamicin, and GYS extract at high (144 mg/mL) or low (36 mg/mL) concentration were placed on the agar surface using sterile forceps and the plates were incubated at 37°C overnight. The bacteriostatic activity was quantified by measuring the diameter of the zone of inhibition around each disc. The data are given as mean ± SEM of three independent experiments.

### Bacteriostatic growth curves

A time-kill kinetics method was used to calculate the bacteriostatic effect of GYS on a panel of target bacteria. GYS extract at 1 × MIC, 2 × MIC, or 4 × MIC (for the respective isolate) was mixed with 3–6 × 10^6^ CFU/mL of bacterial suspension to be tested in a 96-well plate. The plate was incubated at 37°C and the A492 was read at time 0, 1, 2, 6, 12, 24 and 36 h of incubation. Also, samples of the bacterial cultures were taken, serially diluted, spread on nutrient agar in triplicate, and incubated overnight at 37°C. Colony counts were done and means ± SEM were calculated. A control with no antimicrobial compound was run, as well as a sterility control. The bacteriostatic kill-curve experiments were run in triplicate.

### 1,1-Diphenyl-2-picrylhydrazyl (DPPH) free-radical scavenging activity

GYS’s capability for scavenging 1,1-diphenyl-2-picrylhydrazyl (DPPH) free radicals was quantitated using a previously published method [33]. In brief, 1.0 mL of GYS at a concentration of 0.5 mg/mL to 3.0 mg/mL, was added to 2 mL of 0.1 mM DPPH in EtOH at RT for 30 min, and the absorbance was read at 517 nm. A vitamin C (Vc) solution (0.20–1.20 mg/mL) was used as positive control. The results are given as percent DPPH radicals scavenged by Eq (1):

$$\text{DPPH}\;\text{radical}\;\text{scavenging}(\%)=\left[1-\left({\text{A}}_{\text{I}}-{\text{A}}_{\text{II}}\right)/{\text{A}}_{\text{III}}\right]\times 100$$ (1)

where A_I_ = sample; A_II_ = anhydrous ethanol solution; A_III `_= blank control.

### Hydroxyl radical scavenging capacity

The hydroxyl radical scavenging capability of GYS was determined using the salicylic acid reaction. Different concentrations of GYS (0.5–3.0 mg/mL) were mixed with 9.0 mM ferrous sulfate, 9.0 mM salicylic acid in anhydrous ethanol, or 8.8 mM hydrogen peroxide. After 30 min at 37°C, the absorbance at 510 nm was read. Vitamin C (0.2–1.2 mg/mL) acted as the positive control. The results are expressed as a percentage of radicals scavenged (Eq 2):

$$\text{Hydroxyl}\;\text{radicals}\;\text{scavenged}\left(\%\right)=\left[1–\left({\text{A}}_{\text{I}}{-\text{A}}_{\text{II}}\right){\text{/A}}_{\text{III}}\right]\times 100$$ (2)

where A_I_ = sample, A_II_ = hydrogen peroxide solution, and A_III_ = blank control.

### Statistical analyses

The data were analyzed and graphed using GraphPad Prism 8.0 and Origin 2021. The data are the means of at least three independent experiments ± SD. To establish whether differences were significant, an unpaired two-tailed Student’s *t* test or one-way ANOVA with Duncan’s multiple comparisons was employed. The 95% significance level was met by *p* ≤ 0.05, while *p* ≤ 0.01 was considered extremely significant.

## Results

### Chemical profiling of GYS

**Fig 1:**
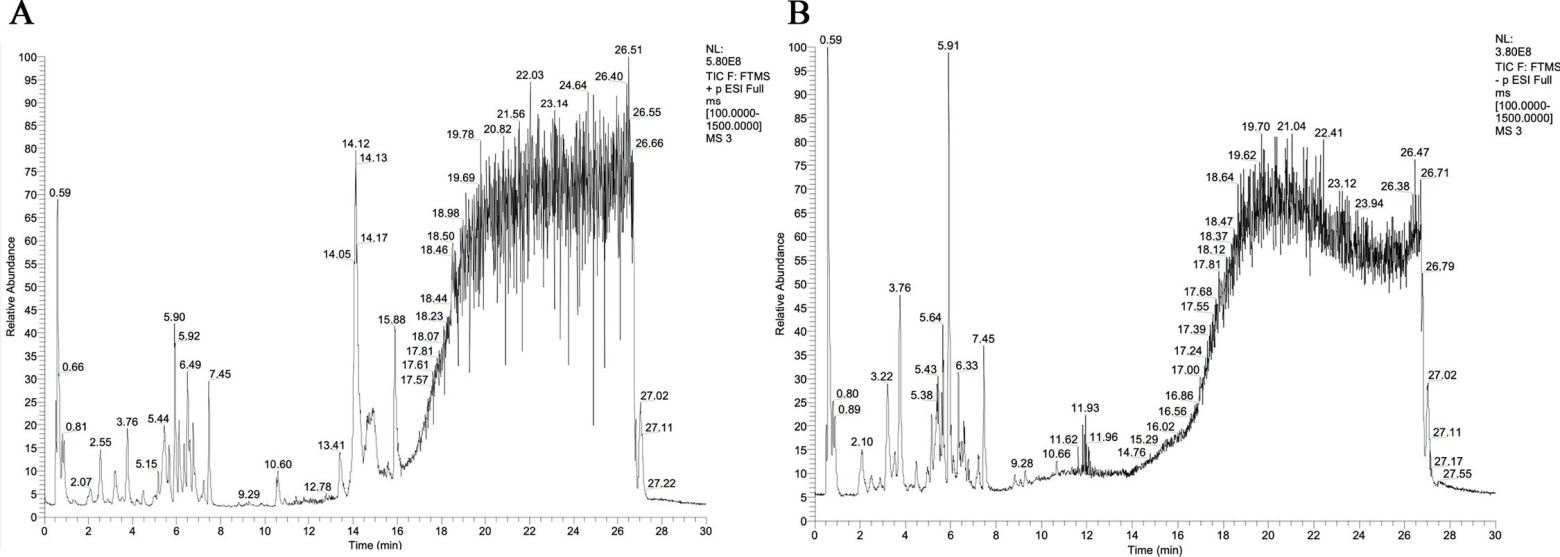
Identification of chemical components of GYS by UHPLC-QE-MS. Total ion chromatography in positive (A) and negative (B) ion modes.

UHPLC-QE-MS analysis was employed to characterize the chemical profile. Seventy-two compounds from GYS were detected and tentatively characterized by the intersection between positive and negative modes (Fig 1). The data for these components is shown in S1 Table. These compounds include different types of constituents, such as phenols, terpenes, alkaloids, organic acids and their derivatives, flavonoids, etc.

### Target prediction and validation of GYS compounds

A total of 446 active GYS compounds were found by reference to the TCMSP and ETCM databases. The ETCM search yielded dandelion and loofah, including 236 in *Lonicerae japonica floss*, 150 in *Forsythiae fructus*, 32 in *Tetrapanacis medulla*, 17 in *Fritillaria thunbergia bulbs*, 9 in loofah and 2 in dandelion. With OB≥30% and DL≥0.18 as the screening criteria, 68 potential active compounds were found. Of the 59 results, 55 active compounds were eligible for further study after the exclusion of duplicates (S2 Table). The 343 genes associated with the identified components were obtained using UniProt databases.

### Acquisition of disease target data and screening of intersecting targets

**Fig 2:**
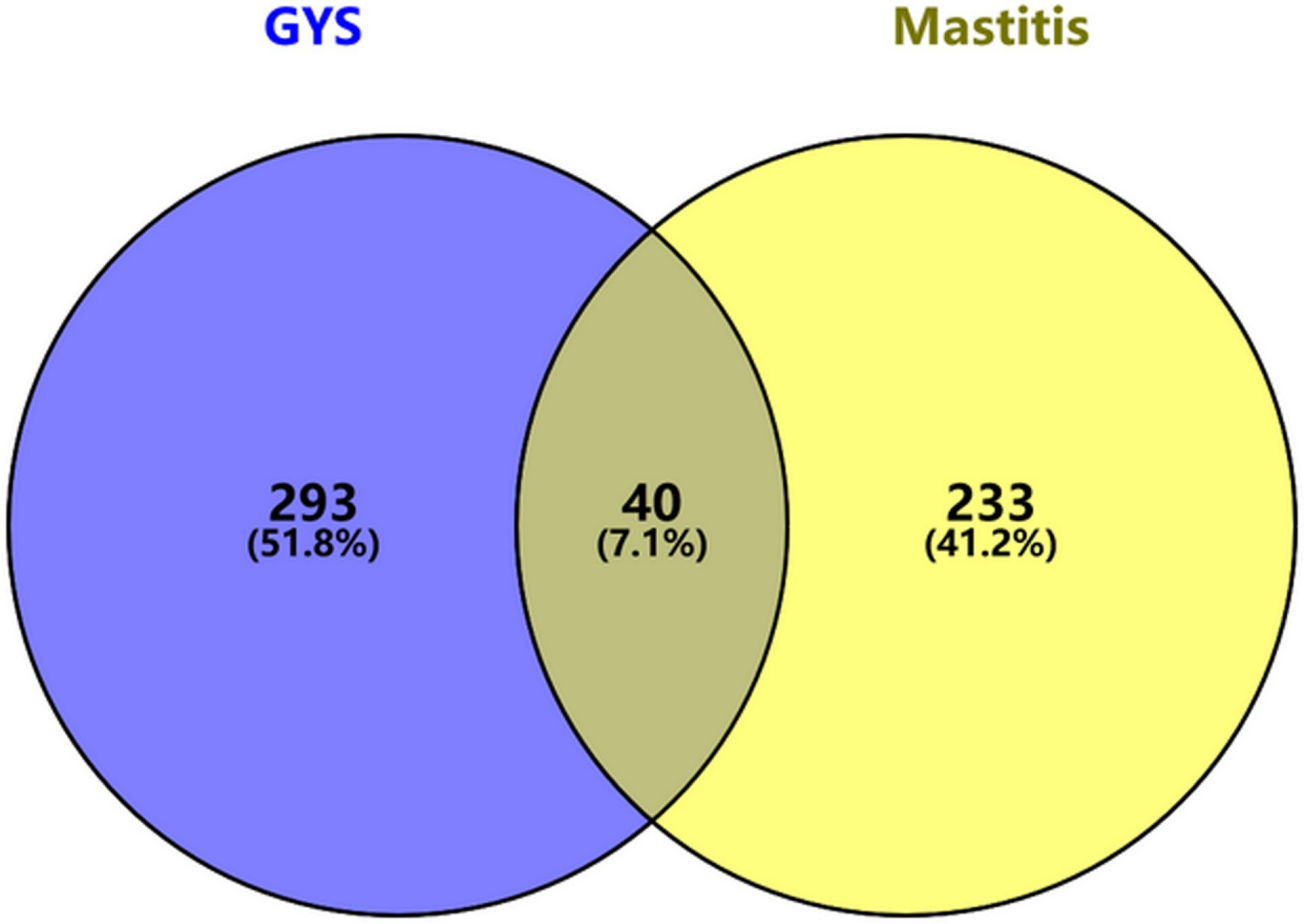
Venn diagram showing the intersection of targets of GYS active components with potential mastitis-associated targets.

Using the DisGENT database, we collected 273 potential mastitis-related GYS targets. Based on this data, we used the Venn Atlas platform to show the forty common intersecting targets (Fig 2).

### Functional enrichment analysis of the active ingredient targets

**Fig 3:**
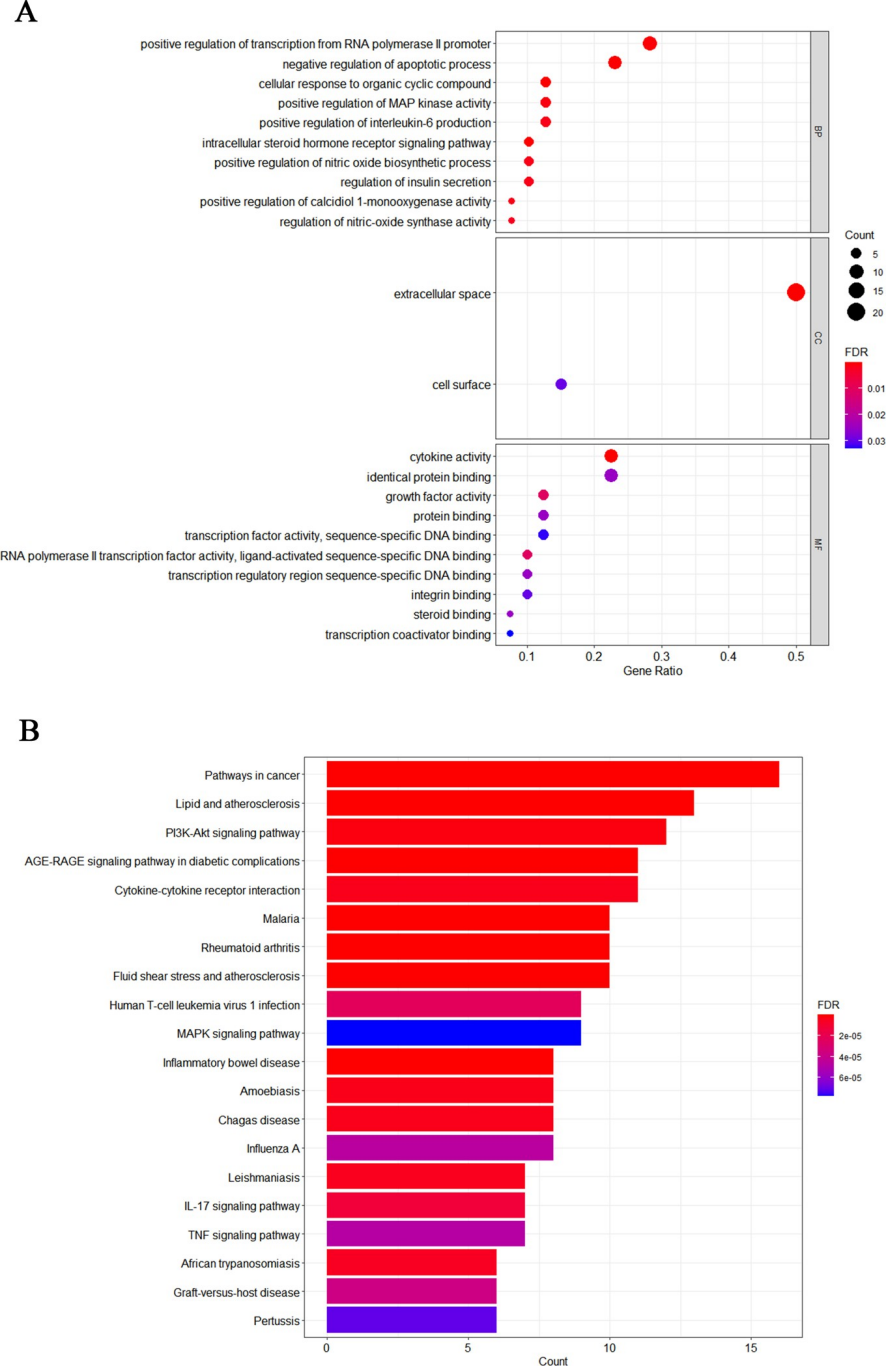
GO and KEGG enrichment analysis for identifying mechanism of the anti-mastitis activity of GYS. (A) Distribution of GO elements in biological processes, molecular functions, and cellular composition (for FDR<0.05). The x-axis shows counts of enriched targets, while the y-axis shows the GO category of the target gene. (B) The top 20 KEGG pathways (FDR<0.05). The color corresponds to *p*-value threshold, and the dot size indicates the number of genes for each term. The x-axis shows the enrichment score, and the y-axis gives the main pathways.

To show enrichment of the anti-mastitis function of GYS, we used 40 overlapping targets. In the GO analysis, all targets were significantly enriched in biological processes (BPs), cellular components (CCs), and molecular functions (MFs) (Fig 3A). The enriched BPs included: positive regulation of transcription from RNA polymerase II promoter; negative regulation of apoptosis; cellular responses to organic cyclic compound and positive regulation of MAPK kinase activity. The extracellular space and the cell surface represented the enriched CCs. In terms of MF enrichment, the identified genes were significantly involved in protein interactions, homologous protein binding, and enzyme binding. The results also showed positive modulation of transcription from the RNA polymerase II promoter, which could positively regulate gene expression.

KEGG enrichment screening of the 40 common targets and top 20 enriched pathways are depicted as bubble plots (Fig 3B) generated with the R program. The results indicate that these targets are highly correlated with cancer-related pathways, and pathways in cancer are the most significantly enriched (Fig 3C). The NF-κB pathway is essential for the development and progression of mastitis, and it was shown to be a likely target for anti-inflammatory and anticancer treatments. Five mastitis-related pathways were found, including the signaling pathways for TNF, MAPK, PI3K-Akt, and IL-17. The AGE-RAGE pathway, which is associated with diabetes severity, was also over-represented. The downstream signals of AGE-RAGE, the p38, and JNK MAPKs altered NF-κB action, affecting the inflammatory response.

### Protein-protein interaction (PPI) network analysis

**Fig 4:**
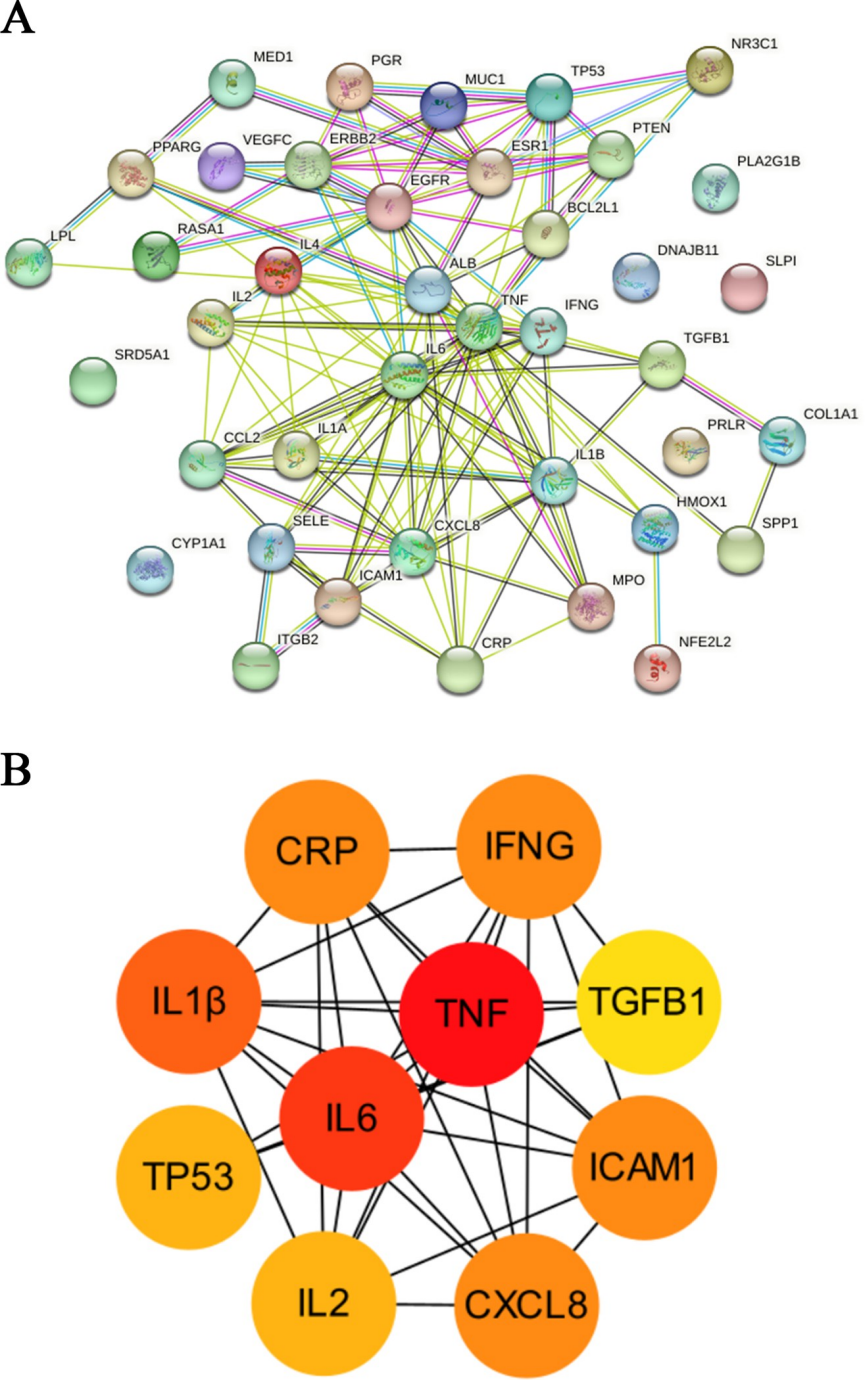
Construction of PPI network and core targets. (A) PPI network showing the relationship among common GYS targets associated with the anti-inflammatory activity. (B) TSV files were obtained from the STRING dbase and imported into Cytoscape 3.8.0 for visualization. The colors represent the importance of the network from most (*red*) to least (*yellow*).

The STRING database was used to construct a PPI network to elucidate the molecular mechanisms of the effects of GYS on mastitis and the activities of specific proteins (Fig 4A). Based on topological analysis, the target genes (degree score ≥16), in order from high to low, were: *TNF*, *IL-6*, *IL-1β*, *ICAM1*, *CXCL8*, *CRP*, *IFNG*, *TP53*, *IL-2* and *TGFB1* (Table 1). These target genes interacted most closely with other targets, and the results suggested that they included the core target (hub) genes. To visualize and identify core targets, we utilized Cytoscape to construct a network based on target degree. We obtained five core targets from this model: TNF, IL-6, IL-1β, ICAM1, and CXCL8 (Fig 4B). These targets probably reflect the principle functional sites of GYS for mastitis therapy and show that GYS influences mastitis via multiple targets.

**Table 1:** Mastitis about the core target hub genes.

Targets	Uniprot ID	Degree	Protein name
TNF	Q06599	14.0	Tumor necrosis factor
IL-6	P26892	13.0	Interleukin-6
IL-1β	P09428	12.0	Interleukin-1 beta
ICAM1	Q95132	9.0	Intercellular adhesion molecule 1
CXCL8	P79255	9.0	Interleukin-8
CRP	P02741	9.0	C-reactive protein
IFNG	P07353	9.0	Interferon gamma
TP53	P67939	8.0	Cellular tumor antigen p53
IL-2	P05016	8.0	Interleukin-2
TGFB1	P18341	7.0	Transforming growth factor beta-1 pro-protein

### Establishment of a comprehensive interaction network of GYS against mastitis

**Fig 5:**
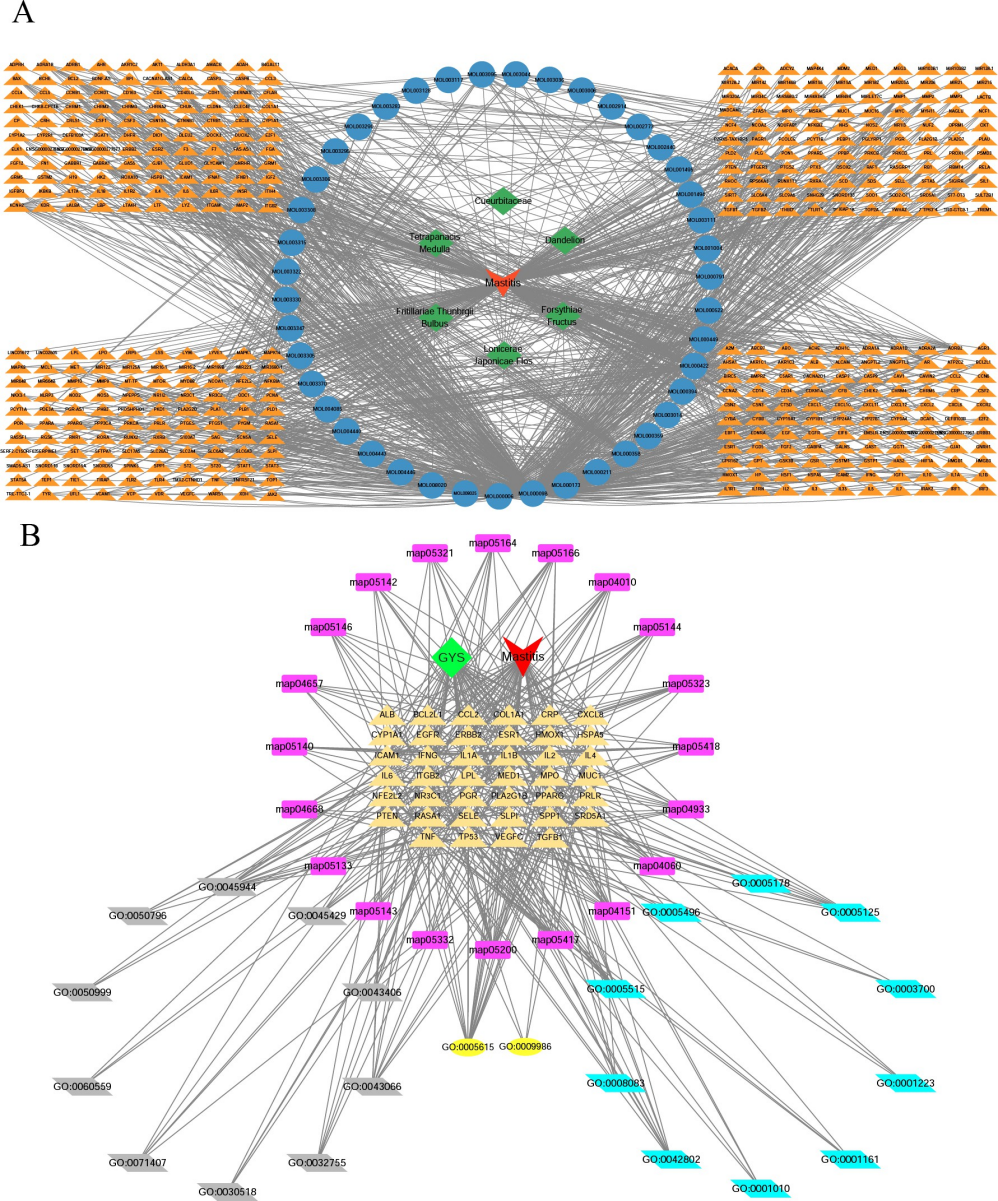
Compound-Disease-Target network and GO, KEGG association network. (A) Network of specific compounds-targets-diseases of the anti-inflammatory ingredients of GYS. The orange nodes show the potential therapeutic targets of GYS against mastitis, and the blue nodes stand for the network’s compounds. The green square nodes show the TCM ingredients, and the red nodes are those associated with mastitis. (B) Interaction networks showing core biotargets, pharmacological functions, and signaling pathways of GYS compounds against mastitis. The yellow nodes show the hub targets of GYS against mastitis, and the nodes in grey are the enriched BPs from hub targets. The yellow ellipse nodes show the enriched CCs and the blue nodes are enriched MFs. The violet nodes show the KEGG enrichment analysis of GYS against mastitis.

The Cytoscape network diagram of C-T-D is shown in Fig 5A. The GYS-mastitis network consists of 497 nodes (448 target nodes, 42 active compound nodes, one inflammatory disease node, and 6 TCM nodes) and 1290 edges; network analyses showed that the average degree of the 48 candidate components was 41.6. The top ten active ingredients based on degree-value are shown in Table 2. Five components had degree values greater than 41.6: quercetin (371), luteolin (146), kaempferol (96), beta-sitosterol (55), and stigmasterol (47), and these were regarded as the key GYS components that are active against mastitis. The GO analyses were used to generate a network showing the relationships between the top 20 pathways and protein-encoding genes (Fig 5B).

**Table 2:** The top ten active compounds in degree-value.

Compound	PubChem CID	Source
Quercetin	5280343	Lonicerae Japonicae Flos, Forsythiae Fructus
Luteolin	5280445	Lonicerae Japonicae Flos, Forsythiae Fructus
Kaempferol	5280863	Lonicerae Japonicae Flos, Forsythiae Fructus
Beta-sitosterol	222284	Lonicerae Japonicae Flos, Forsythiae Fructus, Fritillariae Thunbrgii Bulbus
Stigmasterol	5280794	Lonicerae Japonicae Flos,
Wogonin	5281703	Forsythiae Fructus
5-hydroxy-7-methoxy-2-(3,4,5-trimethoxyphenyl)chromone	10970376	Lonicerae Japonicae Flos,
Beta-carotene	5280489	Lonicerae Japonicae Flos,
Bicuculline	10970376	Forsythiae Fructus
Taraxasterol	115250	Dandelion

### Molecular docking results

**Fig 6:**
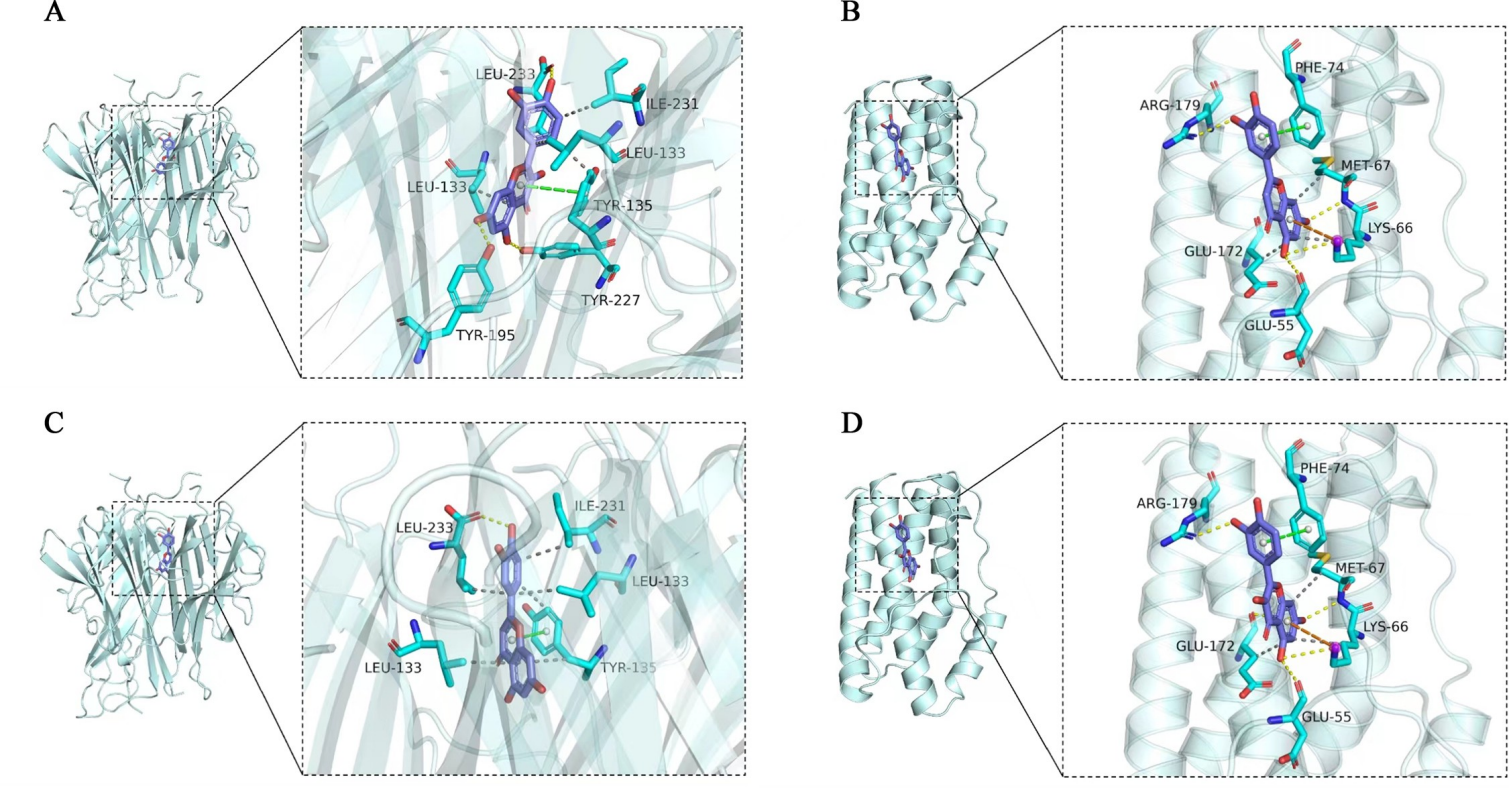
The protein ligands of the docking simulation. (A) Docking of quercetin with TNF. (B) Docking of quercetin with IL-6. (C) Docking of luteolin with TNF. (D) Docking of luteolin with IL-6.

Molecular-docking analysis was performed to confirm that TNF and IL-6 bound to quercetin and luteolin, respectively. Quercetin forms four H-bonds with TNF at Leu233, Leu133, Tyr227 and Tyr195 (Fig 6A). Five H-bonds were detected between quercetin and IL-6 (Fig 6B), and one H-bond between luteolin and TNF (Fig 6C). Luteolin and IL-6 were linked with three H-bonds at Arg179, Met67, Lys66 and Glu55 (Fig 6D). The binding energies were measured to determine the degree of complementarity between the active compounds and the proteins.

Low binding energy indicates greater stability. The binding energy of TNF and quercetin, TNF, and luteolin were −8.8 and −9.1 kJ/mol, respectively. The binding energies of IL-6, quercetin, IL-6, and luteolin were −6.7 and −6.7 kJ/mol, respectively (Table 3). The results indicated that the compounds bound to the active sites of the targets.

**Table 3:** Binding energy of active compounds with core-targets.

Targets	Compounds	
	Quercetin	Luteolin
TNF	-8.8 kcal/mol	-9.1 kcal/mol
IL-6	-6.7 kcal/mol	-6.7 kcal/mol

### Antimicrobial activity of GYS extract

**Fig 7:**
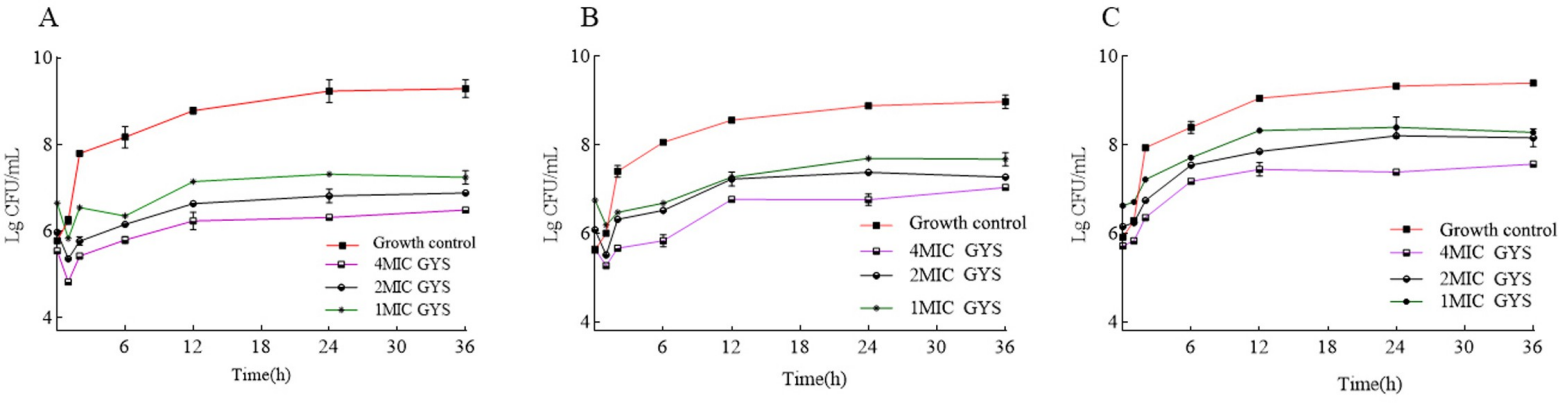
Time-dependent bacteriostatic growth curve of GYS on (A) *S*. *aureus* ATCC 29740, (B) *S*. *agalactiae* CVCC 3940 and (C) *E*. *coli* CVCC 1450.

The MIC and MBC values of GYS against *S*. *aureus*, *E*. *coli*, and *S*. *agalactiae* are shown in Table 4. GYS’ strongest inhibitory effect was on *S*. *aureus* (CVCC 2257), while growth of *E*. *coli* (CVCC 1450) was only slightly affected. The antimicrobial activities of GYS extract against *S*. *aureus*, *E*. *coli*, and *S*. *agalactiae* are tested with the bacteriostatic circle approach (Table 5). The high concentrations of GYS diameters of the antibacterial ring are 24.04 ± 0.20, 15.07 ± 0.19, and 18.11 ± 0.59 mm against *S*. *aureus*, *E*. *coli*, and *S*. *agalactiae*, respectively, which are between vancomycin (17.01 ± 0.16 mm) and cephalexin (25.07 ± 0.15 mm). The Time bacteriostatic curve of GYS against the three pathogenic pathogens has a better inhibitory effect on Gram-positive bacteria (*S*.*agalactiae* CVCC 3940, *S*. *aureus* CVCC 2257) than on Gram-negative bacteria (*E*. *coli* CVCC 1450). The Lg CFU/mL value reduces bacteria within one hour, and the Lg CFU/mL value increases slowly after two hours (Fig 7).

**Table 4:** Antimicrobial activity of GYS extract against common bacterial species.

Bacteria	MIC (mg/mL)	MBC (mg/mL)
*S*. *aureus* CVCC 2257	4.50	9.00
*E*. *coli* CVCC 1450	18.00	36.00
*S*. *agalactiae* CVCC 3940	9.00	18.00

**Table 5:** Bacteriostatic activity of GYS extract against common bacterial species.

Diameter of inhibition zone (mm)	*S*. *aureus* CVCC 2257	*E*. *coli* CVCC 1450	*S*. *agalactiae* CVCC 3940
Penicillin	/	—	/
Cephalexin	25.07 ± 0.15	/	31.02±0.17
Gentamicin	/	24.05 ± 0.29	/
Vancomycin	17.01 ± 0.16	/	25.06 ± 0.18
GYS 1 (36 mg/mL)	11.22 ± 0.54	—	7.02 ± 0.26
GYS 2 (144 mg/mL)	22.04 ± 0.20	15.07 ± 0.19	18.11 ± 0.59

"—" indicates that there is no bacteriostatic zone, "/" indicates that the test was not run.

### *In Vitro* antioxidant activity

**Fig 8:**
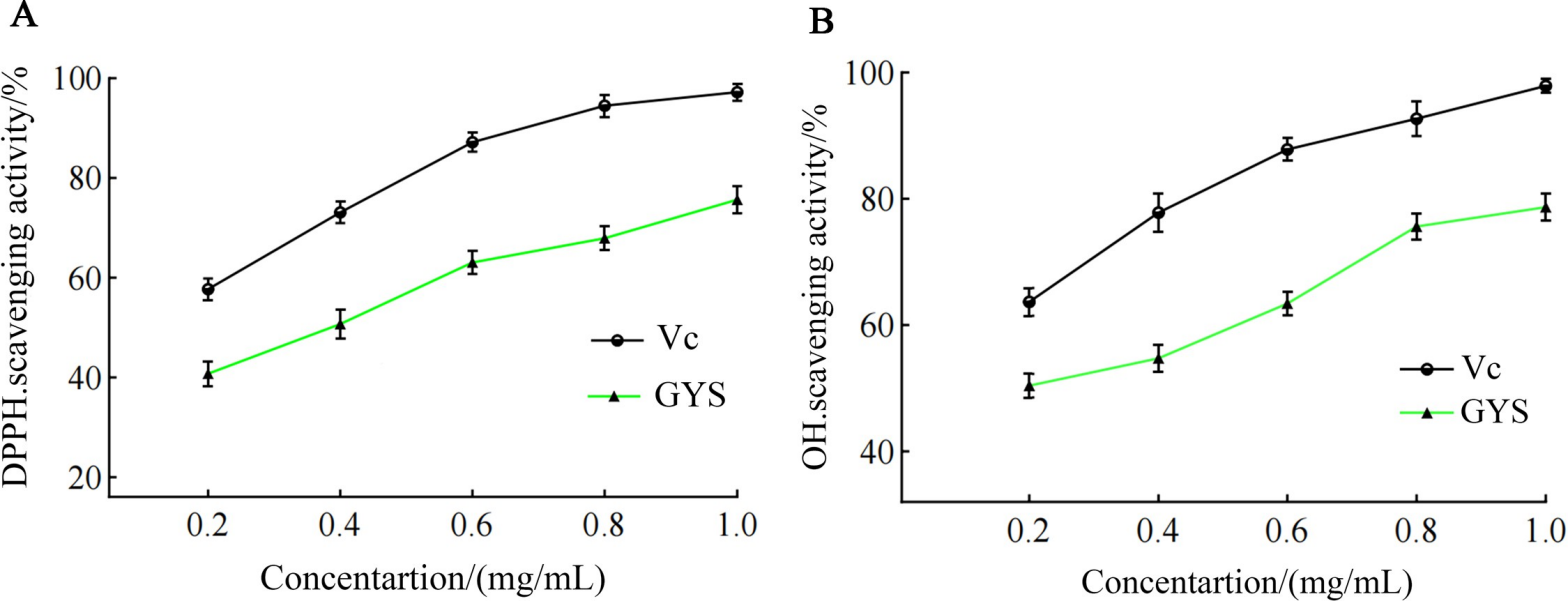
Antioxidant activity of the GYS extract *in vitro*. (A) DPPH radical scavenging assay. Assays were run in triplicate in three independent experiments. (B) The •OH-scavenging activity of GYS extract compared with Vc (vitamin C) as positive control.

The antioxidant action of GYS in vitro were investigated by DPPH free-radical scavenging (Fig 8A). The DPPH scavenging ability of the GYS extract was lower than that of the positive control (Vc). Both were concentration-dependent and in the range of 0.20–1.00 mg/mL. The GYS concentration capable of 50% removal of DPPH radicals (IC50) was 0.38 mg/mL by GYS, while for vitamin C it was 0.15 mg/mL. However, at 1.00 mg/mL, the •OH-scavenging activity of GYS was 78.70 ± 2.17%. The •OH-scavenging activity (IC50) of GYS was 0.24 mg/mL and for Vc it was 0.06 mg/mL (Fig 8B).

## Discussion

The Chinese traditional medicine formulation, Gong Ying San has been used as a mastitis treatment for many years [34, 35]. The *Dandelion*, *Lonicera japonica flos*, and *Forsythia fructus* in this recipe are cooling and thus detoxify the body, disperse wind, and reduce heat. Several other herbs include *loofah*, *Medulla tetraparesis*, and *Fritillaria thunbergia bulbs*, which ventilate milk, reduce swelling and disintegrate [11]. However, the mechanism of GYS’s pharmacological action on mastitis has yet to be completely worked out. Here, we performed a comprehensive evaluation using non-targeted metabolomics, network pharmacology, and molecular-docking assays to delineate the underlying mechanisms and therapeutic targets of GYS in mastitis. The antibacterial inhibitory zone measurements, antibacterial growth curve plotting, and assays for DPPH and hydroxyl free radical scavenging experiments verified the antibacterial and antioxidant capability of GYS.

The primary active compounds in GYS were identified by UHPLC-QE-MS. We identified 40 compounds, including phenols, terpenes, alkaloids, organic acids and their derivatives, flavonoids, etc., that contain some antibacterial properties. *Streptococcus spp*., *Corynebacterium spp*., and *Enterococcus spp*., *S*. *aureus spp*. arre colonizers of skin and mucosa, and the primary cause of bovine mastitis [36]. Research showed that mammary gland infection decreased antioxidant capacity of the milk and reduced the activity of catalase, lactoperoxidase, glutathione peroxidase, and superoxide dismutase, and the level of antioxidant vitamins [37]. Flavonoids exert antibacterial effects by active synergy against bacterial virulence factors, such as quorum-sensing signal receptors, enzymes, and toxins [38]. However, enhanced antioxidant activity may be the primary mechanism, including binding pro-oxidant iron, scavenging reactive nitrogen, chlorine, and oxygen species, and potentially inhibiting cyclooxygenases and lipoxygenases [39]. When bovine mastitis occurs, decreased KEAP1 and increased Nrf2, HO1, NQO1, and reactive oxygen species (ROS) indicate increased oxidative stress [40]. Thus, the presence of the isolated antioxidant compounds may explain why GYS extract reduces inflammation and has other anti-mastitis properties.

Using our network pharmacology model, we identified 55 active ingredients in GYS. Quercetin, luteolin, kaempferol, beta-sitosterol, and stigmasterol were the main active components in GYS for mastitis treatment as they affected the most mastitis-related symptoms. Quercetin is a flavonol widely distributed in the plant kingdom with multiple biological activities, including maintaining normal bone metabolism and slowing the progression of inflammatory disease [41]. It has been reported that quercetin can be used as a non-antibiotic therapy for mastitis by inhibiting NF-κB transcription in the Nrf2-ARE signaling pathway [42], which aligns with our results on the anti-inflammatory properties of GYS. Our results show that quercetin reduces mastitis by regulating the TNF signaling pathway [43]. The antioxidant luteolin showed potent anti-inflammatory activity and inhibited mast cell activation when administered with the anti-inflammatory cytokine IL-38. IL-38 acts through a different pathway, and significantly increased the observed anti-inflammatory effect [44]. Kaempferol, a natural polyphenol, reduced T-cell proliferation, and nitric oxide release, and inhibited ROS formation. Xiang [45] reported that kaempferol down-regulated the mRNA expression of *TNF-α* and *IL-6* in a murine model of non-alcoholic steatohepatitis induced by a high-fat diet and exhibited excellent antioxidant and anti-inflammatory activities [46]. Beta-sitosterol showed in vivo anti-inflammatory and antioxidant activities. Previous tests of beta-sitosterol showed a specific antibacterial ability and significant reduction in apoptosis, oxidative stress, and inflammation by activation of the hypoxia-inducible factor-1(HIF-1a) / mammalian target of rapamycin (mTOR) signal pathway [47, 48]. These findings suggest that beta-sitosterol can potentially reduce mastitis-induced inflammatory responses in mammary glands. Studies have shown that stigmasterol inhibited the infiltration of inflammatory cells and the over-secretion of mucus. The anti-inflammatory effects resulted from a decrease in IL-6 and NF-κB, and an increase in antioxidant activity [49]. The active components of GYS were found to bind to core proteins, and this was verified by molecular-docking analyses. Thus, GYS’ inflammation-lowering activity resulted from inhibiting the expression of proinflammatory cytokines.

Previous studies demonstrated that luteolin could suppress inflammation mediated by IL-1β, IL-6, IL-8, IL-17, IL-22, TNF-α and COX-2, which act through the NF-κB, JAK–STAT, and TLR pathways [50]. In addition, luteolin exhibited a potentially effective new function by protecting tissues from mastitis damage caused by *S*. *aureus* infection [51]. Kaempferol suppressed phosphorylation of the NF-κB p65 subunit and the degradation of its inhibitor, IκBα, in the NF-κB signal pathway for the treatment of mastitis; this was accompanied by decreased myeloperoxidase (MPO) production and *ANGPTL2* expression, as well as a reduction in TNF-α and IL-6 [52]. The TNF-α-induced inflammation corresponded to an increase in ICAM-1 mRNA and protein, significantly inhibited by kaempferol treatment in A549 cells [53].

In summary, the main GYS ingredients targeted multiple functions, and many overlapping targets were found for each of the principle components, indicating that the activity of GYS could be amplified through synergy between its compounds. These studies support the hypothesis that GYS can effectively treat mastitis by eliminating inflammation through the synergistic interaction of its bioactive components. However, as the interaction between the various GYS components plays an essential anti-mastitis role, it still needs to be determined if specific doses and proportions of quercetin, luteolin, and kaempferol would have anti-mastitis effects similar to GYS.

GO enrichment screening showed that the effectiveness of GYS therapy was primarily a result of its influence on the inflammatory response through the processes of apoptosis, hypoxia, and cellular response to LPS. In the present study, most of the proteins involved were distributed in the extracellular space, and the primary molecular functions included protein binding, enzyme binding, and cytokine activity. Moreover, using PPI network analysis, we determined that TNF, IL-6, IL-1β, CXCL8, and ICAM1 proteins were the main targets of GYS for mastitis treatment. Previous reports emphasized the central role of TNF-α as a regulator of immunity, and confirmed that the TNF-α signaling pathway was essential for regulating the inflammatory response [54, 55]. In line with our hub gene identification, studies have reported that the cytokines TNF-α and IL-6 were most frequently associated with the inflammatory response. TNF-α increased the secretion of IL-6 and other cytokines and exacerbated mastitis-induced tissue damage through excess ROS [56]. Additionally, according to our KEGG pathway results, eleven pathways were significantly enriched. We found that GYS mainly interfered with mastitis through the PI3K-Akt signal pathway [57], the MAPK pathway [56], the TNF signaling [58], the AGE-RAGE pathway, and the fluid-shear stress and atherosclerosis pathways, which are closely related to the reported pathology of mastitis [59, 60].

The KEGG enrichment report revealed that the AGE-RAGE pathway was another player in the anti-mastitis activity of GYS, as it was correlated with the progression of mastitis in diabetic complications [61]. Additional investigations showed significant anti-inflammatory effects of AGE-RAGE inhibition in diabetic complications [61, 62]. Notably, the three target genes, *COL1*, *MMP2*, and *SELE*, in AGE-RAGE signaling can all be affected by quercetin [63], which suggests that quercetin may be the most promising GYS compound for mastitis therapy. The AGE-RAGE pathway in diabetes plays a vital role in developing mastitis, including the expression and release of proinflammatory cytokines causing tissue destruction [64].

It was also claimed that the downstream PI3K/Akt signal transduction pathway can be activated by AGE and RAGE. The PI3K/Akt pathway is crucial for growth, survival, metabolism, proliferation, transcription, and protein synthesis [65]. The study revealed that the PI3K/Akt pathway altered the expression of tight-junction proteins, protected the integrity of the blood-milk barrier, and reduced murine mastitis [66]. Intriguingly, GYS was also beneficial in other inflammatory diseases, such as intrauterine adhesion and suppurative otitis media [67, 68]. The inflammatory response to intrauterine adhesions can increase IL-6 and TNF-α in menstrual effluent in the uterus [18], and GYS treatment significantly reduced expression of the pro-inflammatory cytokines in rats with endometritis [69]. Our molecular docking analysis revealed that TNF and IL-6 were primary targets for interaction with the active GYS constituents, quercetin, and luteolin, against mastitis. The results revealed that the binding energy between GYS components and relevant targets was less than -5 kcal/mol [70], which suggested that TNF and IL-6 might be the primary targets of GYS in mastitis treatment. Thus, GYS may be one of the best TCM formulations for treating inflammatory conditions involving elevated TNF and IL-6 levels.

In animal metabolism, the production and elimination of ROS requires a dynamic balance [71]. The enzymatic defense system is one of the two systems with high free-radical scavenging capability. It is comprised principally of superoxide dismutase (SOD), catalase (CAT), and glutathione peroxidase (GSH-Px), which effectively remove free radicals and terminate free radical chain reaction [72]. Zhao et al. [73] found that the body’s defensive antioxidant capacity against free radicals was closely related to overall health status. Yin et al. [74] found that the SOD, GSH-Px, and CAT activity in the serum of cows with mastitis were significantly inhibited compared with the control group. It is speculated that the decreased antioxidant activity in the serum of diseased cows results in the accumulation of free radicals, and more severe pathological outcomes of mastitis. Jin et al. [75] treated cows with latent mastitis with anti-inflammatory lactation granules (composed of dandelion, honeysuckle vine, forsythias, etc.), and showed a dramatic decrease in MDA content compared to control treatment. At the same time, T-AOC and SOD were significantly increased. Thus, dandelion, honeysuckle, and other traditional Chinese medicines have an excellent scavenging effect on MDA produced in the body and can improve SOD activity and T-AOC ability of the body to a certain extent. Improving the body’s antioxidant capacity indicates that the damaged mammary gland is gradually recovering [76].

Regarding limitations, some target genes and compounds may have been excluded from the public database because of the nature of the network pharmacology methodology. Although we identified quercetin, luteolin, kaempferol, beta-sitosterol, and stigmasterol as the active components of GYS, their comparability to GYS might be lacking. Additional in vivo and in vitro research must be done to test this hypothesis.

Our study was the first to identify the primary components, targets, and pathways of GYS responsible for its anti-inflammatory effects using network pharmacology and experimentation in a comprehensive manner. The data support the use of GYS as an effective add-on therapy for treating mastitis-related diseases.

## Conclusions

In this paper, network pharmacology and molecular-docking analyses showed that quercetin, luteolin, and kaempferol were the main active components of GYS in mastitis treatment. The key targets, including TNF, IL-6, IL1β, ICAM1, and CXCL8, were obtained using Cytoscape and verified by molecular docking, which supported their essential role as anti-inflammatory agents. The identified targets were intimately associated with physiological functions such as inflammation, positive gene expression and apoptosis regulation, and the responses to hypoxia and LPS. GYS appears to act against mastitis primarily via the TNF and PI3K-Akt signaling pathways. This research establishes a strong foundation for further investigation into the anti-inflammatory effects of GYS and enhances our understanding of the underlying mechanisms.

## Supporting information

S1 Table Characterization of chemical constituents in GYS by UHPLC-QE-MS analysis (figshare database). https://doi.org/10.6084/m9.figshare.25323277.v1.
(DOCX)

S2 Table List of active GYS components. DOI: 10.5061/dryad.sxksn039n (figshare database). https://doi.org/10.6084/m9.figshare.25323280.v1.
(DOCX)

S1 Data (CSV)

S2 Data (XLSX)
