# Supplementary material for: Evaluation of the mechanism of Gong Ying San activity on dairy cows mastitis by network pharmacology and metabolomics analysis
Source: PLoS One. 2024 Apr 17;19(4):e0299234. doi: 10.1371/journal.pone.0299234 (PMC11023200; doi:10.1371/journal.pone.0299234)
Supplement: S1 Table — https://doi.org/10.6084/m9.figshare.25323277.v1. (DOCX) [file pone.0299234.s001.docx]

**Evaluation of the mechanism of** **Gong Ying San activity on dairy cows mastitis by** **network pharmacology and** **metabolomics analysis**

**Supplementary information**

**Supplementary Table 1.** **Characterization of chemical constituents in GYS by** **UHPLC-QE-MS analysis.**

| Name | Formula | InChIKey | Class | RT(min) | Quasi-Molecular Ion(m/Z) |
| --- | --- | --- | --- | --- | --- |
| 3,4,5-trimethoxycinnamic acid | C12H14O5 | YTFVRYKNXDADBI-SNAWJCMRSA-N | Phenylpropanoids | 2.48 | 256.1154885 |
| Rosmarinic acid | C18H16O8 | DOUMFZQKYFQNTF-WUTVXBCWSA-N | Phenylpropanoids | 2.95 | 361.0901692 |
| Cryptochlorogenic acid | C16H18O9 | GYFFKZTYYAFCTR-AVXJPILUSA-N | Phenylpropanoids | 3.41 | 355.1020252 |
| (E)-3-[4-[(2S,3R,4S,5S,6R)-3,4,5-trihydroxy-6-(hydroxymethyl)oxan-2-yl]oxyphenyl]prop-2-enoic acid | C15H18O8 | LJFYQZQUAULRDF-FDGSXQGBSA-N | Phenylpropanoids | 3.42 | 349.0891014 |
| Chlorogenic acid | C16H18O9 | CWVRJTMFETXNAD-JUHZACGLSA-N | Phenylpropanoids | 4.18 | 355.1021606 |
| Picroside I | C24H28O11 | XZGPUOQGERGURE-LUVHZPKESA-N | Phenylpropanoids | 4.34 | 493.1691893 |
| Sinapoylhexoside | C17H22O10 | XRKBRPFTFKKHEF-UHFFFAOYSA-N | Phenylpropanoids | 4.45 | 387.2013699 |
| (1R,3R,4S,5R)-1,3,4-trihydroxy-5-[(E)-3-(4-hydroxyphenyl)prop-2-enoyl]oxycyclohexane-1-carboxylic acid | C16H18O8 | BMRSEYFENKXDIS-QHAYPTCMSA-N | Phenylpropanoids | 4.46 | 339.107228 |
| Calceolarioside A | C23H26O11 | UHIGZYLCYRQESL-VJWFJHQPSA-N | Phenylpropanoids | 4.95 | 479.15406 |
| (4S,5Z,6S)-4-(2-methoxy-2-oxoethyl)-5-[2-[(E)-3-phenylprop-2-enoyl]oxyethylidene]-6-[(2S,3R,4S,5S,6R)-3,4,5-trihydroxy-6-(hydroxymethyl)oxan-2-yl]oxy-4H-pyran-3-carboxylic acid | C26H30O13 | JGHUOJAZXGSFRI-HOWDAYCMSA-N | Phenylpropanoids | 5.59 | 551.1746001 |
| Caffeic acid | C9H8O4 | QAIPRVGONGVQAS-DUXPYHPUSA-N | Phenylpropanoids | 5.64 | 181.0495951 |
| Calceolarioside B | C23H26O11 | LFKQVVDFNHDYNK-FOXCETOMSA-N | Phenylpropanoids | 5.91 | 479.1535037 |
| Acanthoside B | C28H36O13 | WEKCEGQSIIQPAQ-IRBNZIFYSA-N | Phenylpropanoids | 6.44 | 603.2059633 |
| Isochlorogenic acid C | C25H24O12 | UFCLZKMFXSILNL-RVXRWRFUSA-N | Phenylpropanoids | 6.57 | 517.1329401 |
| Ferulic Acid | C10H10O4 | KSEBMYQBYZTDHS-HWKANZROSA-N | Phenylpropanoids | 7.00 | 195.065403 |
| Arctiin | C27H34O11 | XOJVHLIYNSOZOO-SWOBOCGESA-N | Phenylpropanoids | 7.5 | 533.2013165 |
| Umbelliferone | C9H6O3 | ORHBXUUXSCNDEV-UHFFFAOYSA-N | Phenylpropanoids | 10.63 | 163.0387644 |
| (2S,3R,4S)-4-(2-{[(2E)-3-(3,4-Dihydroxyphenyl)-2-propenoyl]oxy}ethyl)-2-(beta-D-glucopyranosyloxy)-3-vinyl-3,4-dihydro-2H-pyran-5-carboxylic acid | C25H30O13 | ZPEFYJBGAZLAKK-CPYBZULYSA-N | Phenylpropanoids | 10.86 | 577.1335008 |
| Gossypetin-8-C-glucoside | C21H20O13 | SJRXVLUZMMDCNG-UHFFFAOYSA-N | Flavonoids | 5.09 | 481.0975386 |
| Quercetin | C15H10O7 | REFJWTPEDVJJIY-UHFFFAOYSA-N | Flavonoids | 5.69 | 303.049445 |
| Biochanin-7-O-glucoside | C22H22O10 | LFEUICHQZGNOHD-UHFFFAOYSA-N | Flavonoids | 5.70 | 447.1293953 |
| Luteolin | C15H10O6 | IQPNAANSBPBGFQ-UHFFFAOYSA-N | Flavonoids | 5.92 | 287.0548526 |
| Afzelin | C21H20O10 | SOSLMHZOJATCCP-AEIZVZFYSA-N | Flavonoids | 5.95 | 433.1124294 |
| Herbacetin | C15H10O7 | ZDOTZEDNGNPOEW-UHFFFAOYSA-N | Flavonoids | 6.10 | 303.0502253 |
| Kaempferol | C15H10O6 | IYRMWMYZSQPJKC-UHFFFAOYSA-N | Flavonoids | 6.20 | 287.0556194 |
| Isorhamnetin | C16H12O7 | IZQSVPBOUDKVDZ-UHFFFAOYSA-N | Flavonoids | 6.30 | 317.0656329 |
| Apigenin-7-O-glucoside | C21H20O10 | KMOUJOKENFFTPU-QNDFHXLGSA-N | Flavonoids | 6.47 | 433.112899 |
| Icariin | C33H40O15 | TZJALUIVHRYQQB-XLRXWWTNSA-N | Flavonoids | 7.75 | 677.2416888 |
| Apigenin | C15H10O5 | KZNIFHPLKGYRTM-UHFFFAOYSA-N | Flavonoids | 8.36 | 271.0599943 |
| Bruceine A | C26H34O11 | LPZSTPCYWWRQFU-VILODJCFSA-N | Terpenoids | 4.36 | 523.2170527 |
| Sweroside | C16H22O9 | VSJGJMKGNMDJCI-ZASXJUAOSA-N | Terpenoids | 4.47 | 381.1158055 |
| Methyl (1S)-7-hydroxy-7-methyl-1-[(2S,3R,4S,5S,6R)-3,4,5-trihydroxy-6-(hydroxymethyl)oxan-2-yl]oxy-4a,5,6,7a-tetrahydro-1H-cyclopenta[c]pyran-4-carboxylate | C17H26O10 | XBGJTRDIWPEIMG-YVAUHRMASA-N | Terpenoids | 5.05 | 391.1606457 |
| Azuleno(5,6-c)furan-1(3H)-one, 4,4a,5,6,7,7a,8,9-octahydro-3,4,8-trihydroxy-6,6,8-trimethyl- | C15H22O5 | MWDNWQAVYQDZQI-UHFFFAOYSA-N | Terpenoids | 6.86 | 283.1540324 |
| 7-hydroxy-1,4a-dimethyl-9-oxo-7-propan-2-yl-2,3,4,4b,5,6,10,10a-octahydrophenanthrene-1-carboxylic acid | C20H30O4 | UFEHYRPBLFGEJW-UHFFFAOYSA-N | Terpenoids | 9.81 | 335.221628 |
| Medicagenic acid | C30H46O6 | IDGXIXSKISLYAC-WNTKNEGGSA-N | Triterpenoids | 10.02 | 503.3379463 |
| 14-hydroxy-14-(hydroxymethyl)-5,9-dimethyltetracyclo[11.2.1.0¹,¹⁰.0⁴,⁹]hexadecane-5-carboxylic acid | C20H32O4 | MRBLTWPEPGRXQN-UHFFFAOYSA-N | Terpenoids | 10.06 | 359.2197712 |
| 4-[2-[(1R,4aS,5R,8aS)-6-hydroxy-5-(hydroxymethyl)-5,8a-dimethyl-2-methylidene-3,4,4a,6,7,8-hexahydro-1H-naphthalen-1-yl]ethyl]-2H-furan-5-one | C20H30O4 | GVRNTWSGBWPJGS-YVKFZJNCSA-N | Terpenoids | 10.45 | 357.2036388 |
| Asiatic acid | C30H48O5 | JXSVIVRDWWRQRT-UYDOISQJSA-N | Terpenoids | 11.35 | 511.3402972 |
| Beta-SITOSTEROL | C29H50O | KZJWDPNRJALLNS-VJSFXXLFSA-N | Terpenoids | 12.62 | 437.3729687 |
| Rhodioloside | C14H20O7 | ILRCGYURZSFMEG-RKQHYHRCSA-N | Phenols | 2.61 | 323.1107169 |
| P-Hydroxybenzaldehyde | C7H6O2 | RGHHSNMVTDWUBI-UHFFFAOYSA-N | Phenols | 3.48 | 123.0440288 |
| Protocatechualdehyde | C7H6O3 | IBGBGRVKPALMCQ-UHFFFAOYSA-N | Phenols | 4.30 | 139.0389189 |
| Epigallocatechin | C15H14O7 | XMOCLSLCDHWDHP-IUODEOHRSA-N | Polyphenols | 5.90 | 307.0816185 |
| Melibiose | C12H22O11 | DLRVVLDZNNYCBX-ABXHMFFYSA-N | Organic oxygen compounds | 0.95 | 365.1055694 |
| L-Tryptophan | C11H12N2O2 | QIVBCDIJIAJPQS-VIFPVBQESA-N | Organoheterocyclic compounds | 2.45 | 205.0973364 |
| syringin | C17H24O9 | QJVXKWHHAMZTBY-GCPOEHJPSA-N | Organic oxygen compounds | 3.65 | 395.1322951 |
| Kojic Acid | C6H6O4 | BEJNERDRQOWKJM-UHFFFAOYSA-N | Organoheterocyclic compounds | 27.08 | 143.0341636 |
| Lamiide | C17H26O12 | VFYACENSDOLJGQ-SNONCDODSA-N | Iridoids | 2.36 | 445.1331407 |
| Swertiamarin | C16H22O10 | HEYZWPRKKUGDCR-QBXMEVCASA-N | Iridoids | 4.20 | 397.1098792 |
| Plumieride | C21H26O12 | AOPMSFXOYJXDNJ-IRFSQMTFSA-N | Iridoids | 5.01 | 471.1504636 |
| Geniposide | C17H24O10 | IBFYXTRXDNAPMM-BVTMAQQCSA-N | Iridoids | 5.10 | 389.1440785 |
| Citric acid | C6H8O7 | KRKNYBCHXYNGOX-UHFFFAOYSA-N | Organic acids and derivatives | 0.78 | 230.9902956 |
| Loganic acid | C16H24O10 | JNNGEAWILNVFFD-CDJYTOATSA-N | Organic acids and derivatives | 2.07 | 377.1439859 |
| Gamma-Linolenic acid | C18H30O2 | VZCCETWTMQHEPK-QNEBEIHSSA-N | Organic acids and derivatives | 9.06 | 279.2319003 |
| Nicotinic acid | C6H5NO2 | PVNIIMVLHYAWGP-UHFFFAOYSA-N | Alkaloids | 0.78 | 124.0391364 |
| L-Valine | C5H11NO2 | KZSNJWFQEVHDMF-BYPYZUCNSA-N | Alkaloids | 5.92 | 118.0861497 |
| Pinoresinol 4-O-glucoside | C26H32O11 | QLJNETOQFQXTLI-WMYFGKAISA-N | Lignans | 6.53 | 538.2283453 |
| (+)-Pinoresinol | C20H22O6 | HGXBRUKMWQGOIE-AFHBHXEDSA-N | Lignans | 8.64 | 359.1485029 |
| 9-hydroxy-10,12-octadecadienoic acid | C18H32O3 | NPDSHTNEKLQQIJ-SIGMCMEVSA-N | Aliphatic acyl | 11.32 | 319.2264941 |
| AZELAIC ACID | C9H16O4 | BDJRBEYXGGNYIS-UHFFFAOYSA-N | Fatty Acyls | 26.47 | 206.1399592 |
| Glutamyltyrosine | C14H18N2O6 | VVLXCWVSSLFQDS-UHFFFAOYSA-N | Carboxylic acids and derivatives | 2.20 | 311.1241822 |
| Glutamylphenylalanine | C14H18N2O5 | XHHOHZPNYFQJKL-UHFFFAOYSA-N | Carboxylic acids and derivatives | 3.25 | 295.1294274 |
| Geniposidic acid | C16H22O10 | ZJDOESGVOWAULF-OGJQONSISA-N | Iridoid glucoside | 3.75 | 397.1102382 |
| Catalposide | C22H26O12 | UXSACQOOWZMGSE-RWORTQBESA-N | Iridoid glucoside | 4.00 | 483.1501908 |
| LPC 16:0 | C24H50NO7P | ASWBNKHCZGQVJV-UHFFFAOYSA-N | Lipids | 13.19 | 496.3400037 |
| (-)-12-hydroxyjasmonic acid | C12H18O4 | RZGFUGXQKMEMOO-BSANDHCLSA-N | Jasmonic acid | 7.99 | 249.1101554 |
| Phellopterin | C17H16O5 | BMLZFLQMBMYVHG-UHFFFAOYSA-N | Coumarins and derivatives | 1.94 | 339.0679535 |
