# Supplementary material for: Evaluation of the mechanism of Gong Ying San activity on dairy cows mastitis by network pharmacology and metabolomics analysis
Source: PLoS One. 2024 Apr 17;19(4):e0299234. doi: 10.1371/journal.pone.0299234 (PMC11023200; doi:10.1371/journal.pone.0299234)
Supplement: S2 Table — DOI: 10.5061/dryad.sxksn039n (figshare database). https://doi.org/10.6084/m9.figshare.25323280.v1. (DOCX) [file pone.0299234.s002.docx]

**Evaluation of the mechanism of** **Gong Ying San activity on dairy cows mastitis by** **network pharmacology and** **metabolomics analysis**

**Supplementary information**

## Supplementary Table 2. **List of active GYS components.**

| [Mol ID](https://old.tcmsp-e.com/tcmspsearch.php?qr=Tetrapanacis%20Medulla&qsr=herb_en_name&token=900fad062b7a84b4f67d2ec6f32f266e) | [Molecule Name](https://old.tcmsp-e.com/tcmspsearch.php?qr=Tetrapanacis%20Medulla&qsr=herb_en_name&token=900fad062b7a84b4f67d2ec6f32f266e) | [OB(%)](https://old.tcmsp-e.com/tcmspsearch.php?qr=Tetrapanacis%20Medulla&qsr=herb_en_name&token=900fad062b7a84b4f67d2ec6f32f266e) | [DL](https://old.tcmsp-e.com/tcmspsearch.php?qr=Tetrapanacis%20Medulla&qsr=herb_en_name&token=900fad062b7a84b4f67d2ec6f32f266e) | Herb |
| --- | --- | --- | --- | --- |
| MOL004443 | Zhebeiresinol | 58.72 | 0.19 | Fritillariae Thunbergii Bulbus |
| MOL001004 | Pelargonidin | 37.99 | 0.21 | Fritillariae Thunbergii Bulbus |
| MOL004446 | 6-methoxyl-2-acetyl-3-methyl-1,4-naphthoquinone-8-O-beta-D-glucopyranoside | 33.31 | 0.57 | Fritillariae Thunbergii Bulbus |
| MOL000358 | Beta-sitosterol | 36.91 | 0.75 | Fritillariae Thunbergii Bulbus |
| MOL004440 | Peimisine | 57.4 | 0.81 | Fritillariae Thunbergii Bulbus |
| MOL001494 | Mandenol | 42 | 0.19 | Lonicerae Japonicae Flos |
| MOL001495 | Ethyl linolenate | 46.1 | 0.2 | Lonicerae Japonicae Flos |
| MOL002707 | Phytofluene | 43.18 | 0.5 | Lonicerae Japonicae Flos |
| MOL002914 | Eriodyctiol (flavanone) | 41.35 | 0.24 | Lonicerae Japonicae Flos |
| MOL003006 | (-)-(3R,8S,9R,9aS,10aS)-9-ethenyl-8-(beta-D-glucopyranosyloxy)-2,3,9,9a,10,10a-hexahydro-5-oxo-5H,8H-pyrano[4,3-d]oxazolo[3,2-a]pyridine-3-carboxylic acid_qt | 87.47 | 0.23 | Lonicerae Japonicae Flos |
| MOL003014 | Secologanic dibutylacetal_qt | 53.65 | 0.29 | Lonicerae Japonicae Flos |
| MOL002773 | Beta-carotene | 37.18 | 0.58 | Lonicerae Japonicae Flos |
| MOL003036 | ZINC03978781 | 43.83 | 0.76 | Lonicerae Japonicae Flos |
| MOL003044 | Chryseriol | 35.85 | 0.27 | Lonicerae Japonicae Flos |
| MOL003059 | Kryptoxanthin | 47.25 | 0.57 | Lonicerae Japonicae Flos |
| MOL003062 | 4,5'-retro-.beta.,.beta.-carotene-3,3'-dione, 4',5'-didehydro- | 31.22 | 0.55 | Lonicerae Japonicae Flos |
| MOL003095 | 5-hydroxy-7-methoxy-2-(3,4,5-trimethoxyphenyl) chromone | 51.96 | 0.41 | Lonicerae Japonicae Flos |
| MOL003101 | 7-epi-vogeloside | 46.13 | 0.58 | Lonicerae Japonicae Flos |
| MOL003108 | Caeruloside C | 55.64 | 0.73 | Lonicerae Japonicae Flos |
| MOL003111 | Centauroside_qt | 55.79 | 0.5 | Lonicerae Japonicae Flos |
| MOL003117 | Ioniceracetalides B_qt | 61.19 | 0.19 | Lonicerae Japonicae Flos |
| MOL003124 | XYLOSTOSIDINE | 43.17 | 0.64 | Lonicerae Japonicae Flos |
| MOL003128 | Dimethylsecologanoside | 48.46 | 0.48 | Lonicerae Japonicae Flos |
| MOL000422 | Kaempferol | 41.88 | 0.24 | Lonicerae Japonicae Flos |
| MOL000358 | Beta-sitosterol | 36.91 | 0.75 | Lonicerae Japonicae Flos |
| MOL000449 | Stigmasterol | 43.83 | 0.76 | Lonicerae Japonicae Flos |
| MOL000006 | Luteolin | 36.16 | 0.25 | Lonicerae Japonicae Flos |
| MOL000098 | Quercetin | 46.43 | 0.28 | Lonicerae Japonicae Flos |
| MOL000173 | Wogonin | 30.68 | 0.23 | Forsythiae Fructus |
| MOL003281 | 20(S)-dammar-24-ene-3β,20-diol-3-acetate | 40.23 | 0.82 | Forsythiae Fructus |
| MOL003283 | (2R,3R,4S)-4-(4-hydroxy-3-methoxy-phenyl)-7-methoxy-2,3-dimethylol-tetralin-6-ol | 66.51 | 0.39 | Forsythiae Fructus |
| MOL003290 | (3R,4R)-3,4-bis[(3,4-dimethoxyphenyl)methyl]oxolan-2-one | 52.3 | 0.48 | Forsythiae Fructus |
| MOL003295 | (+)-pinoresinol monomethyl ether | 53.08 | 0.57 | Forsythiae Fructus |
| MOL003305 | PHILLYRIN | 36.4 | 0.86 | Forsythiae Fructus |
| MOL003306 | ACon1_001697 | 85.12 | 0.57 | Forsythiae Fructus |
| MOL003308 | (+)-pinoresinol monomethyl ether-4-D-beta-glucoside_qt | 61.2 | 0.57 | Forsythiae Fructus |
| MOL003315 | 3beta-Acetyl-20,25-epoxydammarane-24alpha-ol | 33.07 | 0.79 | Forsythiae Fructus |
| MOL000211 | Mairin | 55.38 | 0.78 | Forsythiae Fructus |
| MOL003322 | FORSYTHINOL | 81.25 | 0.57 | Forsythiae Fructus |
| MOL003330 | (-)-Phillygenin | 95.04 | 0.57 | Forsythiae Fructus |
| MOL003344 | β-amyrin acetate | 42.06 | 0.74 | Forsythiae Fructus |
| MOL003347 | Hyperforin | 44.03 | 0.6 | Forsythiae Fructus |
| MOL003348 | Adhyperforin | 44.03 | 0.61 | Forsythiae Fructus |
| MOL003365 | Lactucasterol | 40.99 | 0.85 | Forsythiae Fructus |
| MOL003370 | Onjixanthone I | 79.16 | 0.3 | Forsythiae Fructus |
| MOL000358 | Beta-sitosterol | 36.91 | 0.75 | Forsythiae Fructus |
| MOL000422 | Kaempferol | 41.88 | 0.24 | Forsythiae Fructus |
| MOL000522 | Arctiin | 34.45 | 0.84 | Forsythiae Fructus |
| MOL000006 | Luteolin | 36.16 | 0.25 | Forsythiae Fructus |
| MOL000791 | Bicuculline | 69.67 | 0.88 | Forsythiae Fructus |
| MOL000098 | Quercetin | 46.43 | 0.28 | Forsythiae Fructus |
| MOL008025 | Tetrapanoside B_qt | 40.93 | 0.79 | Tetrapanacis Medulla |
| MOL008020 | Paryriogenin I | 45.26 | 0.79 | Tetrapanacis Medulla |
| MOL008006 | Paryriogenin A | 41.41 | 0.76 | Tetrapanacis Medulla |
| MOL000359 | Sitosterol | 36.91 | 0.75 | Tetrapanacis Medulla |
| MOL001506 | Squalene | 33.55 | 0.42 | Cueurbitaceae |
| MOL000394 | Choline | 0.47 | 0.01 | Dandelion |
| MOL004085 | Taraxasterol | 8.19 | 0.74 | Dandelion |
